# Facing the Coronavirus Pandemic: An Integrated Continuing Education Program in Taiwan

**DOI:** 10.3390/ijerph18052417

**Published:** 2021-03-02

**Authors:** Ting-Fang Chiu, Dachen Chu, Sheng-Jean Huang, Mengju Chang, Yining Liu, Jason Jiunshiou Lee

**Affiliations:** 1 Department of Pediatrics, Taipei City Hospital Zhongxiao Branch, Taipei 115, Taiwan; dai38@tpech.gov.tw; 2 Department of Health and Welfare, University of Taipei, Taipei 100, Taiwan; dad57@tpech.gov.tw (D.C.); daw15@tpech.gov.tw (S.-J.H.); 3 Department of Education and Research, Taipei City Hospital, Taipei 106, Taiwan; z0755@tpech.gov.tw (M.C.); z0858@tpech.gov.tw (Y.L.); 4 Institute of Public Health, National Yang Ming Chiao Tung University, Taipei 112, Taiwan; 5 Department of Neurosurgery, Taipei City Hospital, Taipei 103, Taiwan; 6 Superintendent Office, Taipei City Hospital, Taipei 103, Taiwan; 7 Department of Surgery, National Taiwan University Hospital, Taipei 100, Taiwan; 8 Department of Family Medicine, Taipei City Hospital Yangming Branch, Taipei 111, Taiwan

**Keywords:** integrated continuing education model, continuing education, COVID-19, healthcare workers, health knowledge, knowledge gaps, online learning, local language

## Abstract

This study aimed to identify knowledge gaps regarding coronavirus disease 2019 (COVID-19) and develop an integrated educational program for healthcare workers. First, we designed and validated ten multiple-choice questions to identify knowledge gaps among healthcare workers. Within one month of the online test and curriculum offering, 5533 staff had completed the test, with a completion rate of 84.97%. There were 2618 healthcare workers who answered the pre-test 100% correctly. Those who did not answer the pre-test 100% correctly took multiple tests after learning through the online teaching materials. Eventually, 5214 staff passed the test (pre-test or post-test with 100% correct answers). The result showed that all staff had a low correct rate for personal protective equipment (PPE) use recommendations. The Infection Control Center conducted training sessions for hospital staff on how to wear protective clothing. Information on the selection and use of PPE for infection prevention was provided, and participants were allowed time to practice and familiarize themselves with the correct way to wear PPE. Moreover, the Department of Education and Research continued updating the online learning materials based on the most important updated peer-reviewed published articles. The attending teaching physicians helped to search, translate, and take notes on articles in the local language (traditional Chinese) for other colleagues to read easily. We expect to increase learning opportunities for healthcare workers, even during uncertain times such as the current coronavirus pandemic through (1) the hospital-wide course announcements, (2) the continuous placement of test questions and learning files on the digital learning platform, (3) the placement of journal highlights in cloud folders, and (4) the use of the digital learning platform on mobile phones accessible outside the hospital.

## 1. Introduction

During the past centuries, the total deaths from acute infectious diseases have been replaced by chronic illnesses because of medicine and public health [1]. Compared with the top ten global causes of death in 2010, there were only lower respiratory infections, diarrheal diseases, tuberculosis remaining in 2016, and the mortality of diarrheal diseases and tuberculosis decreased by almost 1.5 million [2]. However, the World Health Organization announced that contagious diseases are now spreading throughout the world much faster due to the highly mobile and interconnected world. The international spread of disease threatens public health security; some examples include pandemic influenza, polio, drug-resistant tuberculosis, and Severe Acute Respiratory Syndrome (SARS) [3]. According to the Taiwan Centers for Disease Control, there were 346 confirmed SARS cases, and 73 of the cases were deceased patients. Ever since, Taiwan’s government and hospitals have gained much experience, established a public health response mechanism, and reformed the hospital infection control strategy [4]. The mechanisms and strategies include (1) developing monitoring stations at harbors and airports in Taiwan, (2) in compliance with the World Health Organization (WHO) hand hygiene campaign, (3) implementing quality improvement projects for infection control inspections in the hospital accreditation system, and (4) acquiring healthcare workers who are pursuing continuing education for infectious diseases every year [4,5,6].

In late 2019, the first patient diagnosed with coronavirus disease 2019 (COVID-19) was from Wuhan, China, and other countries identified a growing number of cases, including the first patient outside China in Thailand (13 January 2020) and the first patient outside Asia in the United States (21 January 2020) [7]. In Taiwan, the first case was diagnosed on 21 January, which was followed by 798 cases by 31 December 2020. Some countries evacuated their citizens from Wuhan by charter flights and quarantined all evacuees for 14 days. The World Health Organization declared on 30 January 2020 that the coronavirus epidemic in China was a public health emergency of international concern. According to the WHO situation report on 13 January 2021, 90,054,813 cases of COVID-19 were confirmed globally [8].

Although Taiwan and China have a complicated relationship, the cross-strait economic ties are strengthened through trade and investments. Many Taiwanese people worked and studied in China and vice versa, so it was difficult for Taiwan to suspend flights to and from China over fears of the virus [9]. This situation makes the control of the pandemic more challenging for the Taiwanese government. Prevention and control measures are essential, particularly the training and preparation of healthcare workers. Since Taiwan had the first case of COVID-19 on 21 January 2020, all hospitals have set up temperature monitoring systems and followed the protocol for prompt recognition and isolation of patients presenting with fever or respiratory illness.

Furthermore, since the end of January, the Taiwanese government has been linking the entry/exit database of the National Immigration Agency to the health insurance database to record the entry/exit time and travel destinations on the Health Insurance Cards of the Taiwanese people who have entered or left Taiwan [10,11]. Hospital staff must check people’s Health Insurance Cards for their recent travel history before allowing them to enter the hospital [11]. For healthcare workers in the clinics and hospitals, the clinical training and continuing education about this emerging infectious disease may hold the potential to improve healthcare-related outcomes.

Taipei City Hospital is a public regional teaching hospital with five general branches and two specialty branches. The area of each branch is spread evenly across Taipei, and all the general branches could provide comprehensive care for Taipei citizens. Among those branches, the HePing branch was the main hospital taking care of the patients with severe acute respiratory syndrome and was sealed off during that period [6,12]. Taipei City Hospital also has the responsibility to participate in infection prevention and control programs and take care of Taipei citizens. Although all healthcare workers must complete at least four hours of continuing education courses per year focusing on infectious diseases, there was a lack of sessions focusing specifically on the emerging contagious disease. Continuing education programs for COVID-19 were mostly held by each specialty. There is limited research focusing on how an institution knows the knowledge gaps among different healthcare workers. After that, the hospital can build an integrated continuing education program to improve the health workers’ ability to protect themselves from infection; diagnose, treat, and care for patients; and keep updating their knowledge during this rapidly changing pandemic era [13]. This study aimed to use validated questionnaires to identify the knowledge gaps experienced by healthcare professionals in different specialties and develop an integrated continuing education model for the duration of the coronavirus pandemic to educate different hospital staff categories on the basics of COVID-19.

## 2. Materials and Methods

This study was a cross-sectional study conducted in February 2020 in Taipei City Hospital. Ten multiple-choice questions (MCQs) were developed to identify and confirm the knowledge gaps of each job category, as well as the professional knowledge and care skills required in the follow-up, through written examinations, which were used to design the follow-up curriculum reinforcement steps. The MCQs underwent two development stages: the generation of items by an infection specialist and expert evaluation. An infection specialist initially designed ten MCQs, which included the basic knowledge of COVID-19 (Q1, Q3), the epidemiology of COVID-19 (Q6), the criteria for reporting suspected cases of coronavirus (Q2, Q5), the timing of hand-washing and the disinfection mechanism of dry hand sanitizers (Q4, Q7), selecting the essential personal protective equipment (PPE) (Q8), and the medical student affairs in the hospital (Q9, Q10) (Table 1). Based on the learning objectives, nine attending teaching physicians assessed items for clarity and relevance on a 4-point ordinal scale. We calculated item-level Content Validation Index (CVI) scores. The overall CVI from the expert review of these ten MCQs was 0.98.

**Figure 1:**
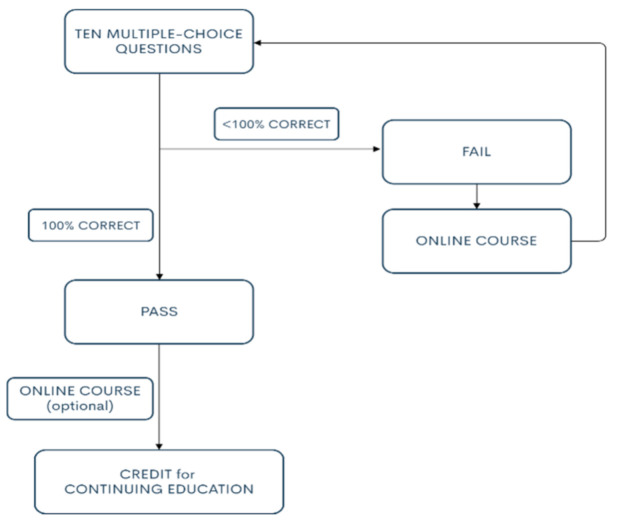
The steps for passing ten online multiple-choice questions about coronavirus disease 2019 (COVID-19).

Then, these ten MCQs were published on the digital learning platform together with the follow-up online learning course and handouts so that information was accessible to all colleagues. In response to the abovementioned ten questions, the Department of Education and Research provided an e-learning course with handouts taught and organized by the infection specialist. The process was monitored until all the participants had completed the multiple-choice questions 100% correctly (Figure 1).

There were 6512 employees in the hospital. Within one month of the online test and curriculum offering, 5533 staff had completed the test, with a completion rate of 84.97%. Four thousand seven hundred twenty-eight healthcare workers responded to the pre-test, and 2618 healthcare workers answered the pre-test 100% correctly. Those who did not answer the pre-test 100% correctly or did not participate in the pre-test could take repeated post-tests after learning through the online teaching materials. Eventually, 5214 staff passed the test (pre-test or post-test with 100% correct answers). To identify the knowledge gaps among different healthcare workers, we classified the employee into three categories: (1) physicians, (2) nurses, and (3) others. The category of physicians included attending physicians and residents, while nurses included nurse practitioners and registered nurses. We categorized clerks, interns, physiotherapists, technicians, and other healthcare workers in the hospital as “others” because they have little chance of direct contact with confirmed or suspected COVID-19 patients. The pre-test respondents’ characteristics, including the gender, age, and length of service time in Taipei City Hospital, were statistically significant within each job category (Table 2). We calculated the passing rate in the pre-test with different cut-off scores for each job category and analyzed the knowledge gaps between different job categories.

The study was conducted according to the Declaration of Helsinki guidelines and approved by the Institutional Review Board of Taipei City Hospital (TCHIRB-10906015-W). This study was conducted by analyzing datasets, and the raw data were de-identified. Therefore, the Research Ethics Committee agreed to waive the informed consent due to minimal risk within the study. All methods were carried out according to the Taipei City Hospital Research Ethics Committee’s relevant guidelines and regulations. All analyses were performed using SAS version 9.4 (SAS Institute Inc., Cary, NC, USA). Chi-square tests were used for categorical variables, and the significance level was equal to or less than 0.05.

## 3. Results

The pre-test passing rate with different cut-off scores for each job category is shown in Table 3. The proportion of physicians answering eight and nine out of 10 questions correct is higher than that of nurses and others, whereas others’ percentage of answering all ten questions correct was 57.15%. Physicians have the highest correct rate for most of the questions, particularly Q6, the epidemiology of COVID-19. In contrast, others have a higher correct rate on the criteria for reporting suspected cases of coronavirus (Q2, Q5). All staff has a low correct rate for Q8, “personal protective equipment recommendations for healthcare workers in the triage area”, which is 76.85% for physicians, 77.28% for nurses, and 76.98% for others (Table 4). After controlling for gender, age, length of service time in Taipei City Hospital, knowledge gaps exist statistically significant between different job categories. Compared with physicians, nurses have higher odds of answering wrong in Q2 (odds ratio (OR): 0.694, 95% confidence interval (CI): 0.505–0.953), Q3 (OR: 0.545, 95%CI: 0.386–0.771), Q5 (OR: 0.596, 95%CI: 0.450–0.789) and Q9 (OR: 0.453, 95%CI: 0.260–0.788), and other healthcare workers have higher odds of answering wrong in Q5 (OR: 0.758, 95%CI: 0.586–0.980), Q7 (OR: 0.596, 95%CI: 0.381–0.933), and Q9 (OR: 0.488, 95%CI: 0.291–0.817). Therefore, the follow-up continuing education curriculum after the test strengthened the physical and online course training on personal protective equipment (PPE) for triage visits and triage areas.

In response to the possibility of healthcare workers being exposed to the virus by caring for suspected patients of COVID-19 or supervising procedures at the hospital entrance, the Infection Control Center conducted training sessions to educate all hospital staff on how to put on and take off protective clothing. The hospital Infection Control Center led the donning and removing PPE training program and provided information on the selection and use of PPE for infection prevention for different situations. The Infection Control Center invited healthcare workers and administrative staff as well as hospital volunteers to participate in the training program because they might also be exposed to cases when assisting people in the following procedures for entering the hospital. The instructors demonstrated how to don and remove PPE correctly, and then, all participants were asked to practice within the proper social distance and received feedback from the instructors. For those who might need detailed instructions or staff who could not attend the training program, the Infection Control Center provided online videos and posters with detailed instructions on putting on and removing PPE. For staff who needed more practice putting on and taking off PPE, the Infection Control Center provided this at each unit’s request. Information on the selection and use of PPE for infection prevention was provided. Participants were allowed time to practice and familiarize themselves with the correct way to don and remove PPE. Given the possible shortage of clinical protective materials, the attending teaching physicians reviewed the timing of and occasions for using surgical masks, N95 masks, and protective clothing. The evidence-based literature was translated into Chinese and made available to the hospital’s decision-makers and colleagues on April 13th to reduce unnecessary use and waste masks.

Taiwan had already taken the lead in controlling the export and sale of masks when the COVID-19 pandemic was anticipated, and the Taiwanese government ensured that hospital staff had enough surgical masks, N95 masks, and isolation gowns to use every day, in addition to the priority distribution of medical supplies [14]. However, there may remain a shortage of medical supplies for relevant hospitals due to the outbreak of COVID-19. Therefore, it was a matter of priority to consider how to use them effectively. The attending teaching physicians reviewed the timing and occasion for using surgical masks, N95 masks, and protective clothing. The evidence-based literature [15,16,17] was translated into traditional Chinese and made available to the hospital’s decision-makers and colleagues, thereby relieving the panic among hospital staff and appreciably reducing the use of N95 masks and isolation gowns in the hospital. The usage amounts of N95 masks in 2020 were 536 per day for 9–15 April, 389 per day for 16–22 April, 325 per day for 23–29 April, 306 per day for 30 April–6 May, and 275 per day for 7–13 May.

Due to epidemics such as SARS and influenza, people in Taiwan had developed good habits such as wearing masks and washing hands frequently. The government set up fever screening stations at the airport, took anti-epidemic actions against human-to-human transmission of COVID-19, and conducted the organization of medical infection control materials timeously. The promotion of epidemic prevention measures was reinforced to cultivate good health habits among the people [14]. All of these, along with the transparency of epidemic prevention policies, ensured that Taiwan’s coronavirus outbreak remained relatively stable. The Department of Education and Research in Taipei City Hospital continued updating online learning materials, which are based on the most important updated peer-reviewed published articles. Nine attending teaching physicians helped to search, translate, and take notes for those articles in the local language (traditional Chinese) for other colleagues to read easily. The attending teaching physicians continued reading and highlighting the latest international COVID-19 literature. From January 24th to mid-July 2020, they had read and summarized more than one hundred essential articles and translated them into traditional Chinese. The easy-to-read Chinese materials were posted on the hospital website, the Taiwan Society of Health-System Pharmacists website, and LINE APP communication software groups (LINE) for reference by relevant medical colleagues nationwide. The attending teaching physicians also categorized the foci of these articles into nine subject areas: epidemiology, diagnostic methods, pathogenic mechanisms, clinical features of adults, clinical features of pediatric patients, clinical features of pregnant women, the latest treatment modalities, epidemic prevention, and ethical issues, hospice care, and teaching. Nine attending teaching physicians created online audio-visual teaching materials using the Pecha Kucha reporting mode (“a fast-paced presentation format consisting of 20 slides set to proceed automatically every 20 s”) [18]. They posted the files onto the digital learning platform in the hospital for those who could not study synchronously.

## 4. Discussion

Taipei City Hospital has had SARS prevention experience for many years and has relevant provisions for infection control as well as education and training. In addition to early activation of the epidemic prevention mechanisms, following national requirements, the Department of Education and Research also conducted online tests among healthcare workers on their basic knowledge of COVID-19, epidemiological knowledge, reporting standards for suspected cases, the timing of hand-washing, and the disinfection mechanism of dry hand sanitizers, recommendations on protective equipment for triage area consultation, and the internship issues concerning medical students. In this way, the department could understand the knowledge gaps in pandemic prevention in different categories and make individualized teaching arrangements for different categories. Compared with previous studies investigating the knowledge gaps between different healthcare professionals [19,20,21], this study revealed that physicians performed better than others among most of the ten basic questions. Nurses were less knowledgeable about administrative procedures, such as reporting suspected cases. The correct rate on the PPE recommendations in the triage area was poor for all categories. Thus, the Department of Education and Research carried out training sessions on administrative circulars for COVID-19, PPE recommendations for triage areas, and other relevant education for different categories.

Moreover, the Infection Control Center was asked to conduct several face-to-face physical training courses on donning and doffing protective equipment, which was filmed and made into videos to illustrate standard operating procedures for colleagues’ reference at any time [22,23]. To encourage the hospital staff to complete this online ten-question test and online reading course, those who answered all ten questions correctly in the pre-test or post-test received one hour of certification for continuing education on emerging infectious diseases in the hospital. To keep up with the ever-changing clinical knowledge of COVID-19, infection specialists and attending teaching physicians additionally designed and validated the second edition of the online test with ten questions and corresponding online learning materials on April 28th, 2020, to continue to provide updates on the correct knowledge for pandemic prevention (Table 5).

Under the protection of national policies, Taipei City Hospital provided each clinical colleague with two surgical masks per day. The emergency department, intensive care unit, and frontline clinical colleagues who dealt with patients suspected of having COVID-19 were provided with N95 masks and protective clothing, which could be replaced in case of contamination. Nevertheless, to bolster the confidence of frontline colleagues in not over-replacing N95 masks and protective clothing and to avoid excessive waste of medical supplies, the attending teaching physicians reviewed relevant domestic and foreign articles to offer online information on the use, storage, and replacement conditions of face shields, masks, and protective clothing, effectively reducing the use of medical materials in Taipei City Hospital [15,16,17].

Although most healthcare workers in Taiwan can read English medical articles, they are undeniably better at reading and quickly understanding the local language (traditional Chinese). When the epidemic information exploded at the early stage of the COVID-19 epidemic, one to two of the most important articles was selected per day, read in detail, and summarized in traditional Chinese by the attending teaching physicians. After being read and proofread by the infection specialists, they were announced online immediately and posted on the hospital network and in the cloud folder for downloading and reading by colleagues. When healthcare workers’ workload increases during the pandemic [24], the ability to read and assimilate the documents quickly was valuable. Due to the good results, colleagues shared the essential files compiled by Taipei City Hospital in the LINE groups for other colleagues to read. The hospital pharmacists also shared those learning files publicly on the Taiwan Society of Health-System Pharmacists’ website. The Joint Commission of Taiwan recognized the unconditional provision of the latest and most important highlighted information in traditional Chinese for proper epidemic prevention by the Taipei City Hospital through the “National Healthcare Quality Award—Epidemic Prevention in Action”. The attending teaching physicians team continued to publish a weekly Chinese update, highlighting one or two essential articles.

The COVID-19 pandemic has had an enormous impact on medical education, and the need for continuous medical education and training is vital in the fight against COVID-19 [25]. The most challenging part includes the use of online technologies among educators and trainees. Although some institutions were hesitant to change their traditional pedagogical approach, the COVID-19 pandemic offered them the best opportunity to shift to online teaching and learning [26,27]. This study can provide information on how medical facilities can quickly conduct online tests for different categories of healthcare workers in the face of emerging infectious diseases such as COVID-19, identify the relative knowledge gaps between different categories, and provide corresponding knowledge reinforcement. More training sessions are needed on the timing of selecting protective equipment and how to put it on and take it off, which seems to be inadequate for all job categories. In times of pandemic information explosion, medical professionals can quickly read the latest medical articles and summarize the local language’s critical points so that healthcare workers who are not native English speakers can quickly absorb and acquire the latest evidence-based medical knowledge. This saves time and provides the most appropriate, evidence-based treatment to the public, under the pandemic’s heavy workload and time constraints.

There were several limitations of this study. First, the pre-test in this study failed to achieve a 100% answer rate or a 100% pass rate, which might be due to the busy workload of clinical staff and the short follow-up period of one month. There was no control group or sampling method in this study, and the results of the MCQs might not be generalizable. In addition, the MCQs’ use has certain limitations, such as time-sensitive questionnaires due to the ever-changing situation with the COVID-19 pandemic, response bias, and lack of understanding. Although the placement of essential journal highlights in the local language in cloud folders was the most useful from the yearly satisfaction surveys, which type of education/training interventions was the most useful needs to be investigated in future studies. Nonetheless, through the hospital-wide course announcements, the continuous placement of test questions and learning files on the digital learning platform, the continuing education course reminder system, the placement of journal highlights in cloud folders, and the use of the digital learning platform on mobile phones, accessible outside the hospital, we expect to be able to increase the learning opportunities for healthcare workers in the hospital, even at a fragmented time, such as that experienced during the coronavirus pandemic

## 5. Conclusions

This study has identified knowledge gaps regarding COVID-19 and developed an integrated educational program for healthcare workers. We designed and validated ten MCQs to identify knowledge gaps among healthcare workers. All healthcare workers needed to be more familiar with adequate wearing the PPE, which is one of the most critical parts of preventing COVID-19. The Department of Education and Research continued updating the online learning materials based on the most essential updated peer-reviewed published articles. The attending teaching physicians helped to search, translate, and take notes on articles in the local language (traditional Chinese) for other colleagues to read easily. We expect to increase learning opportunities for healthcare workers, even during uncertain times such as the current coronavirus pandemic through (1) the hospital-wide course announcements, (2) the continuous placement of test questions and learning files on the digital learning platform, (3) the placement of journal highlights in cloud folders, and (4) the use of the digital learning platform on mobile phones accessible outside the hospital.

## Figures and Tables

**Table 1:** COVID-19 online test (pre-/post-test) 1-Feb-2020 Version.

Q1	The epicenter of the 2019 novel coronavirus (2019-nCoV) is:
	(A) Beijing, China (B) Guangzhou, China (C) Shanghai, China (D) Wuhan, China
Q2	Which category has the Taiwan Centre for Disease Control listed severe Specific Contagious Pneumonia as a statutory infectious disease since 15-Jan-2020?
	(A) First category (B) Second category (C) Third category (D) Fourth category (E) Fifth category
Q3	According to the current evidence, which of the following is the transmission route of the 2019 novel coronavirus (2019-nCoV)?
	(A) Airborne and close contact (B) Droplet and close contact (C) Droplet and airborne (D) Droplet and fecal-oral
Q4	Which of the following is wrong?
	(A) Alcohol dry hand sanitizer cannot kill the virus (B) Environmental cleaning can be done with chlorine bleach (C) Wet hand washing for 40–60 s (D) Wet hand washing for 20–30 s
Q5	For the case definition of Severe Specific Contagious Pneumonia, which of the following is NOT a condition?
	(A) Clinical condition (1): Fever (≥38 °C) with an acute respiratory infection (B) Clinical condition (2): Clinical, radiological, or pathological diagnosis of pneumonia (C) Test condition (1): Clinical specimens (such as throat wipes, sputum, or lower respiratory tract extracts) are isolated and identified with novel coronavirus (D) Test condition (2): The novel coronavirus molecular biology nucleic acid test of clinical specimens is positive
Q6	The epidemiological criteria for Severe Specific Contagious Pneumonia refer to those who have been to Hubei Province, China, within how many days, or have a history of travel to China.
	(A) Seven days (B) Ten days (C) Fourteen days (D) Twenty-one days.
Q7	To protect oneself, which of the following about five opportunities for hand hygiene is wrong?
	(A) Before contact with the patient (B) After contact with the patient (C) After removal of personal protective equipment (D) Before cleaning the environment
Q8	Personal protective equipment recommendations for healthcare workers in the triage area in response to Severe Specific Contagious Pneumonia do not include the following?
	(A) Surgical masks (B) N95 mask and above (C) Gloves (D) Isolation gowns (E) Eye protection equipment
Q9	Which of the following situations does not require suspension of the internship?
	(A) Confirmed contacts within 14 days in health units (B) Entering Taiwan from China, Hong Kong, and Macau within 14 days, such as a travel history in Hubei Province (including Wuhan) (C) Entering Taiwan from China, Hong Kong, and Macau within 14 days, such as a travel history in Guangdong Province (D) Accidental exposure to influenza in a patient without fever
Q10	Personal protection should be done during the internship in the hospital, excluding:
	(A) Washing hands (B) Putting on a mask after entering the hospital (C) Reporting any discomfort to the supervisor (D) The hospital informing the school if the internship is to be suspended

**Table 2:** Characteristics of the pre-test respondents (*N* = 4728).

Demographic Variables	Physicians *N* = 501	Nurses *N* = 1655	Others *N* = 2572	*p*-Value
Gender				<0.001
Male (N, %)	321, 64.07%	52, 3.14%	572, 22.24%	
Female (N, %)	180, 35.93%	1603, 96.86%	2000, 77.76%	
Age (mean ± S.D.)	44.37 ± 10.94	37.74 ± 10.22	43.62 ± 11.17	<0.001
Length of service time in Taipei City Hospital (mean ± S.D.)	11.71 ± 10.19	10.64 ± 9.27	9.59 ± 8.76	<0.001

**Table 3:** Percentage of passing rate of the ten COVID-19 multiple-choice questions with different cut-off scores of different healthcare workers in the pre-test.

Job Categories	Physicians (*N* = 501)			Nurses (*N* = 1655)			Others (*N* = 2572)	Total (*N* = 4728)
	Attending Physicians (*N* = 388)	Residents (*N* = 113)	Total	NursePractitioners (*N* = 107)	Registered Nurses (*N* = 1548)	Total	Total	Total
Percentage of correct answers ≥ 80%	328 (84.54%)	96 (84.96%)	424 (84.63%)	91 (85.05%)	1194 (77.13%)	1285 (77.64%)	2039 (79.28%)	3748 (79.27%)
≥90%	280 (72.16%)	86 (76.11%)	366 (73.05%)	76 (71.03%)	1006 (64.99%)	1082 (65.38%)	1790 (69.60%)	3238 (68.49%)
100%	208 (53.61%)	66 (58.41%)	274 (54.69%)	58 (54.21%)	816 (52.71%)	874 (52.81%)	1470 (57.15%)	2618 (55.37%)

**Table 4:** Knowledge gaps in the ten COVID-19 multiple-choice questions of different healthcare workers.

Category		Physicians (*N* = 501) Correct Numbers %		Nurses (*N* = 1655) Correct Numbers %		Others (*N* = 2572) Correct Numbers %		*p* Value
The basic knowledge of COVID-19	Q1	497	99.20%	1642	99.21%	2544	98.91%	0.571
	Q3	448	89.42%	1347	81.39%	2199	85.50%	<0.001
The epidemiology of COVID-19	Q6	501	100.00%	1624	98.13%	2519	97.94%	0.006
The criteria for reporting suspected cases of coronavirus	Q2	433	86.43%	1339	80.91%	2135	83.01%	0.013
	Q5	406	81.04%	1201	72.57%	1961	76.24%	<0.001
The timing of hand-washing and the disinfection mechanism of dry hand sanitizers	Q4	462	92.22%	1478	89.31%	2324	90.36%	0.145
	Q7	475	94.81%	1590	96.07%	2387	92.81%	<0.001
Selecting the essential personal protective equipment	Q8	385	76.85%	1279	77.28%	1980	76.98%	0.967
The medical student affairs in the hospital	Q9	481	96.01%	1540	93.05%	2392	93.00%	0.040
	Q10	436	87.03%	1466	88.58%	2272	88.34%	0.633

**Table 5:** COVID-19 Online Test Advanced Questions 28-Apr-2020 Version.

Q1	For the government’s tracking and management mechanism for people at risk of infection, who should be quarantined at home?
	(A) Asymptomatic with a travel history in endemic areas within 14 days (B) Contacts of confirmed cases (C) Foreign travelers with fever or respiratory symptoms (D) Foreign travelers with an abnormal sense of smell and taste, or unexplained diarrhea
Q2	For the general guidelines for social networking published by the Taiwan CDC, which of the following is wrong?
	(A) Whether you wear a mask or not, you should maintain a distance of 1.5 m indoors and 1 m outdoors in quiet wind (B) Staff who will come into contact with non-specified people must wear masks throughout the process (C) For open counters that need to face the public, it is recommended to modify or temporarily place transparent partitions (D) People are forced to use alcohol to disinfect their hands at entrances
Q3	Which is the preferred test tool for the 2019 novel coronavirus (2019-nCoV) infection?
	(A) Virus culture (B) Serum antibody test (C) Nucleic acid molecule test (RT-PCR) (D) Electron microscope image
Q4	For the inspection report of a COVID-19 case, which of the following is wrong?
	(A) Increase in the total number of white blood cells (B) Decrease in lymphocytes (C) Possible prolongation of prothrombin time (PT) (D) Possible increase in LDH
Q5	As defined in the report of COVID-19 (COVID-19), the symptoms of clinical conditions do not include which of the following?
	(A) Fever (B) Cough (C) Vomiting (D) Shortness of breath (E) Abnormal sense of smell and taste
Q6	According to current medical literature, the rate of mild COVID-19 is about
	(A) 90% (B) 80% (C) 70% (D) 60%
Q7	Regarding community monitoring, reporting, collection, inspection, and case handling procedures, which of the following is wrong?
	(A) Healthcare workers with fever or respiratory symptoms can be reported to “others” in the forensic system and return to work after 14 days of self-management after a negative result (B) COVID-19 cases that do not require hospitalization after screening can go home for self-management (C) Screening and hospitalization should be performed in a negative pressure room or a separate ward or space (D) Wearing a mask when going home after a case inspection, and the use of public transportation is forbidden.
Q8	What is wrong with the description of novel coronavirus?
	(A) The survival time on plastic surfaces is shorter than that on copper products (B) Infected people can infect others when they are asymptomatic (C) After some confirmed cases develop antibodies in the body, the virus can still be isolated from the respiratory tract specimens (D) Healthcare workers taking care of confirmed or suspected cases should wear N95 masks.
Q9	According to Taiwan CDC’s recommendations, how long can N95 masks be used without contamination?
	(A) 2 h (B) 4 h (C) 6 h (D) 8 h
Q10	Taiwan’s epidemic prevention measures against COVID-19 have been published in international journals, with highlights including:
	(A) The health insurance card is integrated with the entry/exit data so that the doctor can get the travel history warning during a patient’s visit (B) Use new technologies such as QR code for risk classification of passenger infections (C) The Central Epidemic Command Centre plays an active role in allocating resources for epidemic prevention and releasing daily news (D) All of the above

## Data Availability

The datasets produced and analyzed during the present study are available from the corresponding author upon reasonable request.
